# Prognostic Significance of Serum Thyroglobulin Levels and Clinical Factors in Assessing the Efficacy of Radioiodine Therapy in Patients With Hyperthyroidism

**DOI:** 10.7759/cureus.113129

**Published:** 2026-07-21

**Authors:** Salih Azabagic, Adna Mujkic, Ilma Nur Azabagic

**Affiliations:** 1 Department of Internal Medicine, University Clinical Center Tuzla, Tuzla, BIH; 2 Department of Histology and Embryology, Faculty of Medicine, University of Tuzla, Tuzla, BIH; 3 Department of Medicine, Faculty of Medicine, University of Tuzla, Tuzla, BIH

**Keywords:** hyperthyroidism, multinomial logistic regression, radioactive iodine (i-131), thyroglobulin, treatment outcome

## Abstract

Introduction: Radioactive iodine (I-131) has been used for decades as a definitive treatment for hyperthyroidism. However, therapeutic response in clinical practice varies considerably among patients.

Objective: This study aimed to evaluate whether pre-treatment serum thyroglobulin (Tg) levels, patient age, and duration of disease could predict treatment outcome.

Methods: A prospective study was conducted on 146 patients with confirmed hyperthyroidism (Graves’ disease, toxic multinodular goiter, or toxic adenoma). Treatment outcome (euthyroidism, hypothyroidism, or persistent hyperthyroidism) was assessed six months after I-131 administration and analyzed using multinomial logistic regression.

Results: Successful treatment was achieved in 118 of 146 patients (80.82%), while hyperthyroidism persisted in 28 patients (19.18%). Each one-unit increase in Tg raised the odds of treatment failure by 1.004 (p < 0.05). Each additional year of age increased the risk by a factor of 1.09 (p < 0.05), and each additional year of disease duration increased the risk by 1.28, though this did not reach statistical significance (p = 0.06).

Conclusion: Pre-treatment thyroglobulin level and older age are independent and statistically significant predictors of poorer outcome, while longer disease duration showed a trend toward significance (p = 0.06). Patients with elevated Tg levels and a prolonged disease history may warrant consideration for higher initial radioiodine activity, pending further validation.

## Introduction

Hyperthyroidism is not a single disease entity but a group of disorders characterized by excessive thyroid hormone production, each with distinct pathophysiological mechanisms. The most common forms are Graves’ disease (diffuse toxic goiter), toxic multinodular goiter (Plummer’s disease), and toxic adenoma [1].

The clinical spectrum ranges from mild thyrotoxicosis to severe, life-threatening manifestations. Prolonged exposure to elevated thyroid hormones can adversely affect nearly every organ system, with cardiovascular complications, particularly atrial fibrillation, cardiomegaly, and heart failure being of greatest concern. Accelerated bone resorption leading to osteoporosis is another important but often underrecognized consequence [1].

Radioactive iodine (I-131) has been a cornerstone of definitive therapy for hyperthyroidism for decades [1,2]. Its mechanism involves selective destruction of hyperfunctioning thyroid tissue by beta radiation, typically resulting in euthyroidism or, more frequently, hypothyroidism that is readily managed with levothyroxine replacement [3].

In everyday practice, however, outcomes are often less predictable. A substantial proportion of patients experience persistent hyperthyroidism after the initial dose, leading to repeated treatments, prolonged disease exposure, and increased risk of complications [4,5]. Therefore, identifying reliable pre-treatment predictors of therapeutic response is of considerable clinical importance [5,6].

Thyroglobulin (Tg) is a large glycoprotein produced exclusively by thyroid follicular cells. Serum Tg levels are consistently elevated in active hyperthyroidism due to increased glandular mass and cellular stimulation. In this prospective study, we investigated whether pre-treatment Tg levels, in combination with patient age and disease duration, could serve as predictors of I-131 therapy outcome using multivariate statistical modeling.

## Materials and methods

This study is part of a larger analysis conducted on the same patient cohort; a companion paper, "Evaluation of Radioiodine (I-131) Therapy Success in Different Etiological Forms of Hyperthyroidism and Analysis of Response According to Administered Dose" (Original Article, Azabagic S, Mujkic A, Azabagic IN, 2026), is pending publication.

This prospective cohort study was conducted at the Department of Nuclear Medicine and the Department of Endocrinology, University Clinical Center Tuzla, Bosnia and Herzegovina, between 2007 and 2016. The Ethics Committee, University Clinical Center Tuzla, Bosnia and Herzegovina, issued approval 1-12-29-1/06. The study included 146 patients with clinically confirmed manifest hyperthyroidism (Graves' disease, toxic multinodular goiter, or toxic adenoma). All patients meeting the inclusion criteria during the study period were consecutively enrolled.

Inclusion criteria included clinically confirmed manifest hyperthyroidism with elevated free triiodothyronine (FT3) and free thyroxine (FT4) levels, suppressed thyroid-stimulating hormone (TSH), and scintigraphic findings consistent with the underlying diagnosis; availability of serum Tg and thyroglobulin antibodies (TgAb) measurements at baseline and at one, three, and six months post-therapy.

Exclusion criteria included incomplete medical documentation, Hashimoto's thyroiditis, subacute thyroiditis, and a cold nodule on scintigraphy.

All patients underwent a standardized diagnostic workup consisting of clinical examination with thyroid palpation, thyroid ultrasound for volume and morphological assessment, and technetium-99m scintigraphy [1]. Laboratory evaluation included measurement of FT3, FT4, TSH, and serum thyroglobulin. Tg samples were obtained immediately before I-131 administration using an immunoradiometric assay (IRMA) and expressed in ng/mL. Anti-TgAb were measured concurrently using the same assay platform to screen for potential interference with Tg measurement. No patients with clinically significant TgAb-related assay interference were identified in this cohort.

Administered I-131 activity was determined according to institutional fixed-dose protocols based on thyroid volume and clinical severity of thyrotoxicosis, following two standardized regimens: a low-dose protocol (5-15 mCi) for patients with smaller nodule volumes, mild hyperthyroidism, or lower clinical risk profiles, and a high-dose protocol (20-25 mCi) for patients with larger nodule volumes, severe thyrotoxicosis, or a need for more rapid metabolic control. All patients discontinued antithyroid medications (thionamides) for 5 to 7 days prior to I-131 administration, in accordance with standard institutional protocol, to ensure optimal radioiodine uptake.

A formal a priori sample size calculation was not performed. The study enrolled all consecutive patients meeting the eligibility criteria during the study period (2007-2016), resulting in a final sample of 146 patients. This sample size was considered adequate for multinomial logistic regression analysis, which conventionally requires a minimum of 10 events per predictor variable.

Potential confounders considered in this study included disease etiology, patient age, disease duration, and administered I-131 activity.

Serum Tg, patient age, and disease duration were selected as the primary independent variables for the multivariable model based on their established biological plausibility as markers of thyroid gland burden and disease chronicity, their consistent availability as part of standard pre-treatment evaluation, and prior literature support for their prognostic relevance in radioiodine therapy outcomes.

Treatment outcomes were assessed six months post-therapy and categorized as euthyroidism, hypothyroidism, or persistent hyperthyroidism. Statistical analysis included the Kolmogorov-Smirnov test for normality assessment and multinomial logistic regression with euthyroidism as the reference category. Independent variables included pre-treatment serum Tg, patient age, and disease duration in years. Odds ratios (OR) with 95% confidence intervals were calculated, and statistical significance was defined as p < 0.05. Statistical analyses were performed using MedCalc for Windows, version 12.2.1.0 (MedCalc Software, Mariakerke, Belgium).

## Results

Table 1 presents the demographic characteristics of the sample, while Table 2 presents the distribution of patients according to age and disease duration.

**Table 1:** Demographic characteristics of the sample.

Form of hyperthyroidism	Males (n)	Males (%)	Females (n)	Females (%)	Total
Graves' disease	12	8.22	62	42.47	74
Plummer's disease	4	2.74	41	28.08	45
Toxic adenoma	5	3.42	22	15.07	27
Total	21	14.38	125	85.62	146

**Table 2:** Age and disease duration.

Parameter	Males	Females
Age - Mean ± SD	52.6 ± 18.3	52.3 ± 13.9
Age - Minimum	29	25
Age - Maximum	87	80
Disease duration (years) - mean	9.0	9.3

Successful treatment (euthyroidism or hypothyroidism) was achieved in 118 of 146 patients (80.82%). Persistent hyperthyroidism was observed in the remaining 28 patients (19.18%). Among successfully treated patients, 33 (27.97%) achieved euthyroidism, while 85 (72.03%) developed hypothyroidism, consistent with the ablative nature of radioiodine therapy.

Multinomial logistic regression analysis showed that serum Tg and patient age had statistically significant independent effects, while disease duration approached but did not reach statistical significance. The results are summarized in Table 3.

**Table 3:** Results of multinomial logistic regression - predictors of I-131 treatment outcome in hyperthyroid patients. I-131, radioactive iodine.

Predictive parameter	Odds ratio (OR)	95% confidence interval (CI)	Statistical significance (p)
Serum thyroglobulin (Tg) – per unit	1.004	1.001 – 1.008	p < 0.05
Patient age – per year	1.090	1.020 – 1.160	p < 0.05
Disease duration – per year	1.280	1.050 – 1.560	p = 0.06

For the sake of model completeness, Table 4 presents the results to assess the predictors of hypothyroidism versus euthyroidism.

**Table 4:** Predictors of hypothyroidism (vs. euthyroidism)

Predictor	Odds ratio (OR)	95% confidence interval (CI)	Statistical significance (p)
Thyroglobulin (Tg) – per unit	1.000	0.996 – 1.004	0.83
Disease duration – per year	0.920	0.754 – 1.124	0.41
Patient age – per year	0.975	0.907 – 1.049	0.49

In the prediction of hypothyroidism relative to euthyroidism, none of the evaluated predictors (pre-treatment Tg, patient age, or disease duration) reached statistical significance (p > 0.05 for all).

This suggests that, although these parameters are predictive of treatment failure (persistent hyperthyroidism), they do not independently determine the likelihood of developing hypothyroidism after therapy.

The overall model was statistically significant (χ²=34.74, df=18, p=0.001), explaining between 41.9% and 51.7% of the variance (Cox & Snell/Nagelkerke R²).

## Discussion

Elevated pre-treatment thyroglobulin emerged as a significant predictor of treatment failure (OR 1.004 per unit, p < 0.05). This is biologically plausible, as high Tg levels reflect a larger, highly active thyroid gland with greater iodine-trapping capacity, which may require a proportionally higher absorbed dose for adequate ablation [5,6].

Increasing age was also associated with poorer outcomes (OR 1.09 per year, p < 0.05), likely due to age-related fibrotic and involutional changes that alter intrathyroidal iodine kinetics [2].

Disease duration also emerged as a notable predictor, with each additional year associated with a 1.28-fold increased risk of treatment failure (p = 0.06), approaching but not reaching conventional statistical significance. Prolonged stimulation leads to histological changes including hyperplasia, focal fibrosis, and tissue heterogeneity, all of which contribute to relative radioresistance [3,5].

The overall treatment success rate of 80.82% observed in our study is consistent with previously reported rates in the literature, which typically range from 70% to 90% depending on the patient population, disease etiology, and administered activity [3,4]. The predictive role of pre-treatment Tg observed here aligns with the broader concept that markers of thyroid gland burden, including volume, uptake, and secretory activity, are among the most reliable indicators of radioiodine therapy outcome [5,6]. Future studies incorporating thyroid volume measurement alongside Tg in multivariate models may further refine the predictive accuracy of pre-treatment assessments.

These findings support the growing emphasis in the literature on individualized radioiodine dosing protocols based on readily available clinical and biochemical parameters, rather than fixed-dose regimens [1,4,7].

The predictive role of age identified in our study is supported by evidence from the literature. Stachura et al. applied multivariate logistic regression to 144 hyperthyroid patients and similarly found age to be a significant independent predictor of treatment outcome, with an OR of 1.06 per year (95% CI 1.025-1.096, p = 0.001) [8]. The magnitude of this effect is comparable to the OR of 1.09 per year observed in the present cohort, suggesting a consistent, reproducible age-related reduction in radiosensitivity across different patient populations and clinical settings.

The observed association between pre-treatment Tg and treatment failure is biologically consistent with the broader finding that markers of thyroid gland burden are key predictors of I-131 therapy outcome. A meta-analysis by Shalaby et al., encompassing 4,822 patients across 18 studies, identified thyroid volume ≥35.77 mL as an independent predictor of I-131 therapy failure, underscoring the principle that a greater functional thyroid mass requires proportionally higher absorbed radiation doses for adequate ablation [9]. Pre-treatment Tg, as a direct product of follicular cell activity and mass, may therefore serve as a practical surrogate for thyroid volume in settings where volumetric assessment is unavailable.

Alfadda et al. reported a success rate of 83% in patients with Graves' disease receiving a single fixed dose of 13-15 mCi, with higher pre-treatment FT3 concentrations identified as an independent predictor of failure [10]. Together with our findings, these results suggest that pre-treatment biochemical parameters including serum Tg, thyroid hormone levels, and disease duration carry meaningful prognostic information that should be systematically incorporated into treatment planning.

Limitations

This study has several limitations that should be considered when interpreting the results. First, several established predictors of radioiodine treatment outcome, including thyroid volume, administered I-131 activity, disease etiology, TRAb status, and prior antithyroid drug therapy, were not included in the multivariable model. The observed association between serum Tg and treatment failure should therefore be interpreted as an independent statistical predictor rather than a fully adjusted, confounder-free effect. Second, patients with Graves' disease, toxic multinodular goiter, and toxic adenoma were analyzed together despite their differing pathophysiology and radiosensitivity, and disease etiology was not adjusted for in the regression model; subgroup-stratified analyses in larger cohorts may help clarify etiology-specific predictive patterns. Third, although anti-thyroglobulin antibodies were measured concurrently with Tg, formal statistical adjustment for antibody-related assay interference was not performed. Fourth, treatment outcomes were assessed at six months post-therapy; longer follow-up may be needed to fully capture the evolution of thyroid function, as hypothyroidism can continue to develop beyond this period. Finally, this was a single-center study with a moderate sample size, and our predictive model has not been externally validated in an independent cohort; these findings should therefore be confirmed in larger, multicenter, prospective studies before being translated into routine clinical dosing decisions.

## Conclusions

Pre-treatment serum thyroglobulin and patient age are reliable, independent predictors of radioiodine therapy outcome in hyperthyroidism, while disease duration shows a trend toward significance (p = 0.06) that warrants further investigation in larger cohorts. Higher Tg levels and older age are associated with an increased likelihood of persistent hyperthyroidism after the first treatment.

All three parameters are routinely obtained during standard pre-therapy evaluation. Their consideration in patient stratification may help improve first-treatment success rates and reduce the need for repeated radioiodine administration, though this potential benefit should be confirmed in future prospective studies.

## References

[1] Ross DS, Burch HB, Cooper DS (2016). 2016 American Thyroid Association guidelines for diagnosis and management of hyperthyroidism and other causes of thyrotoxicosis. Thyroid.

[2] Campennì A, Avram AM, Verburg FA (2023). The EANM guideline on radioiodine therapy of benign thyroid disease. Eur J Nucl Med Mol Imaging.

[3] Bonnema SJ, Hegedüs L (2012). Radioiodine therapy in benign thyroid diseases: effects, side effects, and factors affecting therapeutic outcome. Endocr Rev.

[4] Metso S, Jaatinen P, Huhtala H, Luukkaala T, Oksala H, Salmi J (2004). Long-term follow-up study of radioiodine treatment of hyperthyroidism. Clin Endocrinol (Oxf).

[5] Allahabadia A, Daykin J, Holder RL, Sheppard MC, Gough SC, Franklyn JA (2000). Age and gender predict the outcome of treatment for Graves' hyperthyroidism. J Clin Endocrinol Metab.

[6] Xing YZ, Zhang K, Jin G (2020). Predictive factors for the outcomes of Graves' disease patients with radioactive iodine (131I) treatment. Biosci Rep.

[7] Stokkel MP, Handkiewicz Junak D, Lassmann M, Dietlein M, Luster M (2010). EANM procedure guidelines for therapy of benign thyroid disease. Eur J Nucl Med Mol Imaging.

[8] Stachura A, Gryn T, Kałuża B, Budlewski T, Franek E (2020). Predictors of euthyreosis in hyperthyroid patients treated with radioiodine (131)I(-): a retrospective study. BMC Endocr Disord.

[9] Shalaby M, Hadedeya D, Toraih EA (2022). Predictive factors of radioiodine therapy failure in Graves' Disease: a meta-analysis. Am J Surg.

[10] Alfadda A, Malabu UH, El-Desouki MI (2007). Treatment of Graves' hyperthyroidism--prognostic factors for outcome. Saudi Med J.

